# Determination of knee cartilage volume and surface area in beagle dogs: a pilot study

**DOI:** 10.1186/s40634-017-0109-1

**Published:** 2017-11-06

**Authors:** Aad Dhollander, Amanda Malone, James Price, Alan Getgood

**Affiliations:** 1 The Fowler Kennedy Sport Medicine Clinic, 3M Centre, The University of Western Ontario, London, ON N6A 3K7 Canada; 2 Eupraxia Pharmaceuticals, Victoria, BC V8R 5J2 Canada; 3 AZ Klina, Department of Orthopedic Surgery and Traumatology, Augustijnslei 100, 2930 Brasschaat, Belgium

**Keywords:** Beagle dogs, MRI, Cartilage, Surface area, Volume

## Abstract

**Background:**

The objective of this study was to determine the cartilage volume and surface area of male and female Beagle dog knees using 3D (3 dimensional) reconstructed MRI images.

**Methods:**

Six Beagle Dogs (Canis familiaris) (3 males and 3 females) of 10-18 months old and weighing between 7.2 and 17.1 kg underwent a MRI evaluation of both knees. The data acquired allowed a 3D reconstruction of the knee and measurement of the cartilage volume and surface area.

**Results:**

Mean knee cartilage volume (averaged over the right and left knees) of animals between 7.2 and 17.1 kg ranged from 319.7 to 647.3 mm^3^; while the mean knee cartilage surface area ranged from 427.14 to 757.2 mm^2^. There was evidence of both knee volume and surface area increasing linearly with animal bodyweight.

**Conclusions:**

The cartilage volume and surface area of the Beagle dog appears to correlate significantly with body weight. This study provides a reference base for future studies investigating cartilage related pathology such as osteoarthritis.

## Background

Osteoarthritis (OA) is a complex disease process involving the whole synovial joint that leads to joint destruction, pain and reduced quality of life. The importance of OA as a chronic health disease is unquestionable. An estimated 27 million individuals are affected with OA in the United States alone with its high prevalence resulting in a significant socioeconomic burden for the nation’s health care industry costing an estimated $185 billion annually (Lawrence et al. 2008; Neogi et al. 2013; Kotlarz et al. 2009). These facts combined with our limited knowledge of OA pathogenesis, underlines the necessity for significant research efforts to enhance our knowledge concerning the disease development and progression. These insights could eventually lead to the development of successful treatment regimens (Kuyinu et al. 2016).

The structural changes within the joint that evolve with OA can take place over a number of years, if not decades, in humans (Matthews 2013). Therefore, it is extremely difficult to precisely study the natural history of changes observed in the early stages of the disease over a shorter time frame. Moreover, the course of this disease is often unpredictable with clinical symptoms often presenting late in the disease process that may not exactly represent the molecular phenomena and structural changes within the joint (Matthews 2013; Haviv et al. 2013; Yusuf et al. 2011). Numerous animal models of OA have therefore been developed over the past 50 years with the aim of surpassing some of these difficulties. Researchers have used these models to enhance their knowledge concerning disease onset and progression, as well as to develop and evaluate new diagnostic tools and therapeutics (McCoy 2015).

Whilst a number of different species have been used, in the last two decades canine models have been developed to examine various aspects of OA (Lahm et al. 2005; Mrosek et al. 2006; Thompson et al. 1991). Anterior cruciate ligament transection, joint surface impact loading and medial meniscectomy models are the most commonly utilized. Although several breeds such as the Labrador, Golden Retriever, and German Shepherd have been incorporated in these models, the Beagle dog is the most commonly used, particularly when studying disease modifying OA drugs (DMOADs) for later regulatory approval.

To be able to test these drugs that will later be translated to human clinical trials for OA, experimental dose levels of new therapies are calculated based on cartilage volumes of the target species and breed. The cartilage volume of the human knee has been demonstrated to be 23.3 cm^3^ (16.6 to 31.4 cm^3^) per joint (Eckstein et al. 2001). Currently, the cartilage volume and surface of Beagle dogs is still unknown, despite their use in many different OA studies. The volume of cartilage in the dog knee has historically been calculated as a proportion of human tissue relative to its weight. This results in volume of 1.1 +/− 0.7 cm^3^ based on a 25.0 kg animal (Boileau et al. 2008).

A more accurate way to determine cartilage volume is to use magnetic resonance imaging (MRI). MRI is capable of visualizing hyaline cartilage noninvasively in vivo enabling the quantification of the cartilage volume by 3D post processing techniques on the basis of the acquisition of MRI data sets (Harada et al. 2011). Semi-automated methods have already been described that achieve 3D segmentation of the articular cartilage (Brem et al. 2009; Bae et al. 2009). These volumes can be used as a direct measure of the response of articular cartilage and its functional adaptation to mechanical stimulation, for example during immobilization and physical training. More specifically, repeated assessments of the cartilage volume in the early stages of degenerative joint disease may be an effective way of monitoring the loss of cartilage tissue during disease progression, and the subsequent effectiveness of disease modifying therapy.

In contrast to local cartilage thickness measurements at one specific point, the assessment of the cartilage volume does not depend on the choice of a specific location, which can be difficult to define in cross-section and to reproduce in longitudinal studies. However, at present no reference values exist for Beagle dogs in the literature as to what should be considered a normal or abnormal cartilage volume. Moreover, the quantitative relationship of the different cartilages within the Beagle dog knee joint (patella, femur, medial tibia, lateral tibia) is unknown, and it is also unclear which anthropometric parameters are associated with their normal variation (Eckstein et al. 1998; Eckstein et al. 2001). The objective of this study was to determine the cartilage volume and surface area of male and female Beagle dog knees using 3D (3 dimensional) reconstructed MRI images.

## Methods

### Study population

Six Beagle Dogs (*Canis familiaris*) (3 males and 3 females) were received from Covance Research Products, Inc. or Marshall Bioresources and were only used for this study. Animals were 10-18 months old and weighed between 7.2 to 17.1 kg at the onset of MRI evaluation. The exact ages of the dogs were 10, 13 and 18 months for the females; 17, 18 and 18 months for the males. Housing and transport of the animals throughout the study was conformed to the guidelines cited in the Guide for the Care and Use of Laboratory Animals and the applicable standard operating procedures of CiToxLAB North America, Inc. The dogs were transferred only once from CiToxLAB North America to the MRI facility. Five days were allowed between transfer and MRI evaluation. Animals were single or group housed in stainless steel dog cages each equipped with an automatic watering system.

### MRI imaging

Cage side clinical observations and mortality checks were performed twice daily throughout the study. A detailed clinical examination was performed and individual body weights were recorded for all animals prior to MRI evaluation by an attending veterinarian. Animals were fasted overnight prior to the MRI evaluation. Animals were anesthetized during scanning using propofol (6 mg/kg, IV) followed by intubation. Additional propofol doses were given when necessary. Lidocaine spray (10% *w*/w) was administered to the glottis prior to intubation when needed. Anesthesia was maintained with isoflurane. An ophthalmic ointment was applied to both eyes to prevent drying of the cornea. Both knees were examined for obvious signs of previous injury, any gross morphologic abnormality or ligamentous laxity. Image acquisition was under the supervision of a certified veterinary neurologist.

**Fig. 1:**
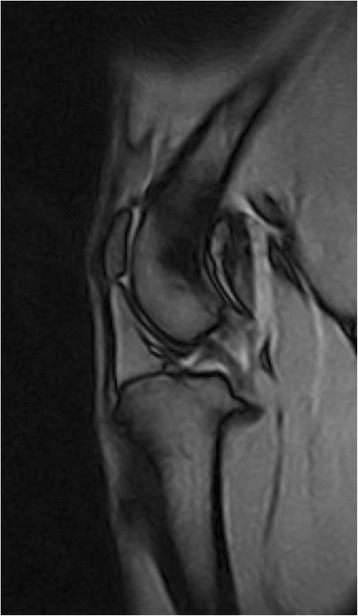
A sagittal intermediate weighted gradient-echo MRI image of a right Beagle dog knee. These images were used to calculate cartilage volumes and surface areas

The MRI sequence consisted of a sagittal intermediate weighted gradient-echo MRI acquisition (Tr/Te: 1600 ms/21 ms, Matrix: 224x224px, FOV: 210x210mm, NEX: 4, ST: 3 mm, Reconstruction: 512x512px) (Fig. 1). This was performed on the dog’s both knees using an Esaote 0.25 T O-scan apparatus (Esaote Canada, Indianapolis, IN).

Cartilage volume was calculated using the previously described technology adapted for the dog (Boileau et al. 2008; Kauffmann et al. 2003). Bone to cartilage and cartilage to soft tissue interface of the cartilage tissue were delineated in the baseline MRI sequence and 3D cartilage object was computed. The quantitative measurement of the cartilage volume and area was performed on the MRI images using Cartiscope (ArthroVision Inc., Montreal, Quebec, Canada) as previously described (Pelletier et al. 2007; Raynauld et al. 2004; Raynauld et al. 2006). The cartilage volume of the entire (total) knee and five subregions was assessed: the trochlea, the lateral and medial femoral condyles, and the lateral and medial tibial plateaus. The patella was excluded from the evaluation. The limit between femoral condyles and trochlea was defined by the natural anatomical structures of the dog.

Cartilage thickness could be estimated based on the surface area and volume calculations.

### Statistical methods

Descriptive graphics relating bodyweight to knee compartment and total volumes and surface areas were created. For the purposes of describing the relationship, a simple linear model was built for each case relating the measured endpoint to bodyweight, with each animal donating 2 observations, one from each knee. The *p*-values for the gradient parameter are also presented, using the same model but using the average of the 2 measurements per animal as the dependent variable. Due to the small sample size, no further compartmentalization of the system variance was performed. Additionally, no attempt was made to look at non-constant residual variance relationships with bodyweight, or curvature in the regression. From these models, 95% prediction and confidence intervals for the regression curves were produced. Where these intervals crossed the zero-line, due to the simple linear relationship, the intervals were truncated at zero. All statistics and graphics were produced using the R software, in particular the packages dplyr and ggplot2 (R Core team 2016; Wickham 2009; Wickham et al. 2016).

## Results

In one animal, the cartilage surface area and volume of the tibial aspect of the knee could not be estimated due to insufficient data to perform adequate 3D reconstruction of this section; this animal does not contribute data to the total knee volume and surface area analyses.

**Fig. 2:**
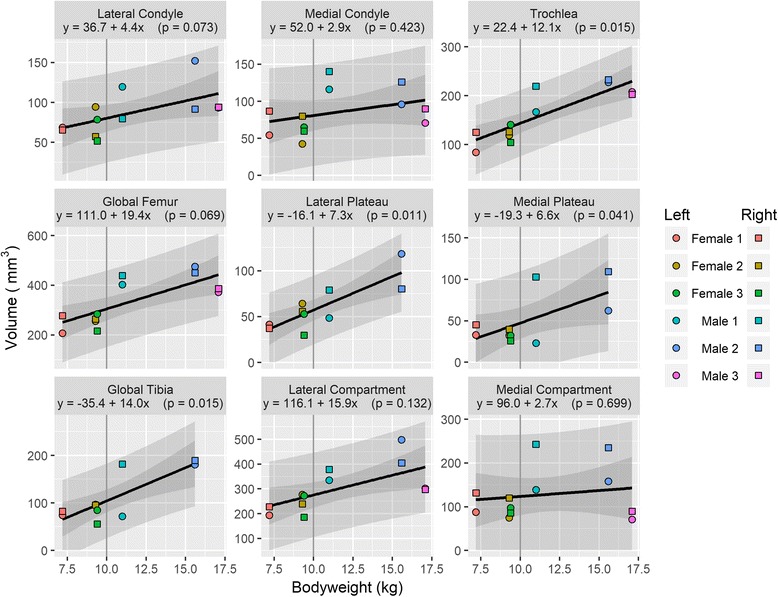
Animal bodyweights regressed against knee compartment volumes. Data are presented from both knees from each animal, where available. The regression equation from a simple linear model of volume versus bodyweight is given in each panel header, along with the *p*-value for the gradient. The lighter shaded region shows the 95% prediction interval for this model, illustrating the range of plausible future values under this model; the darker shaded region shows the 95% confidence interval for the regression line

**Fig. 3:**
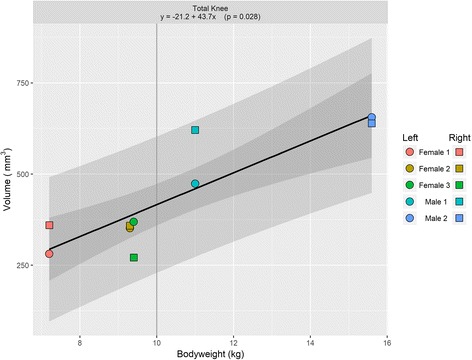
Animal bodyweights regressed against total knee volume. Data are presented from both knees from each animal, where available. The regression equation from a simple linear model of volume versus bodyweight is given in each panel header, along with the p-value for the gradient. The lighter shaded region shows the 95% prediction interval for this model, illustrating the range of plausible future values under this model; the darker shaded region shows the 95% confidence interval for the regression line

**Fig. 4:**
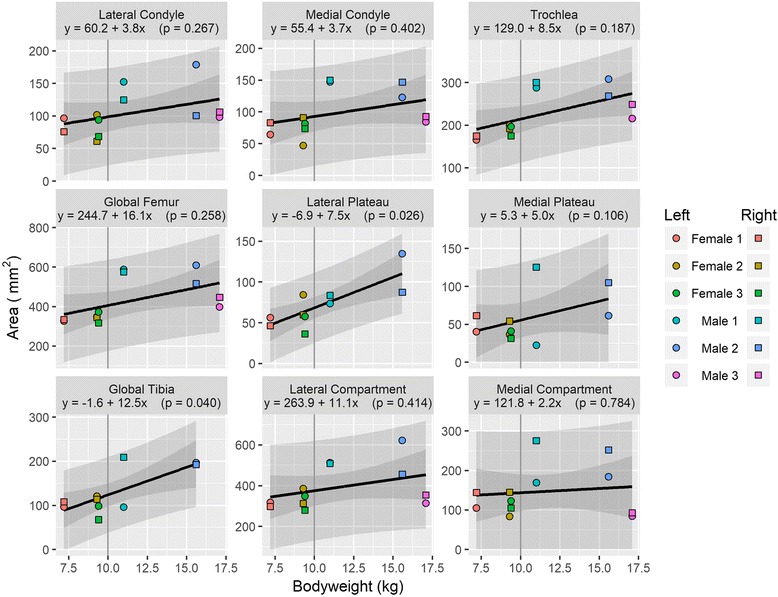
Animal bodyweights regressed against knee compartment surface areas. Data are presented from both knees from each animal, where available. The regression equation from a simple linear model of surface area versus bodyweight is given in each panel header, along with the p-value for the gradient. The lighter shaded region shows the 95% prediction interval for this model, illustrating the range of plausible future values under this model; the darker shaded region shows the 95% confidence interval for the regression line

**Fig. 5:**
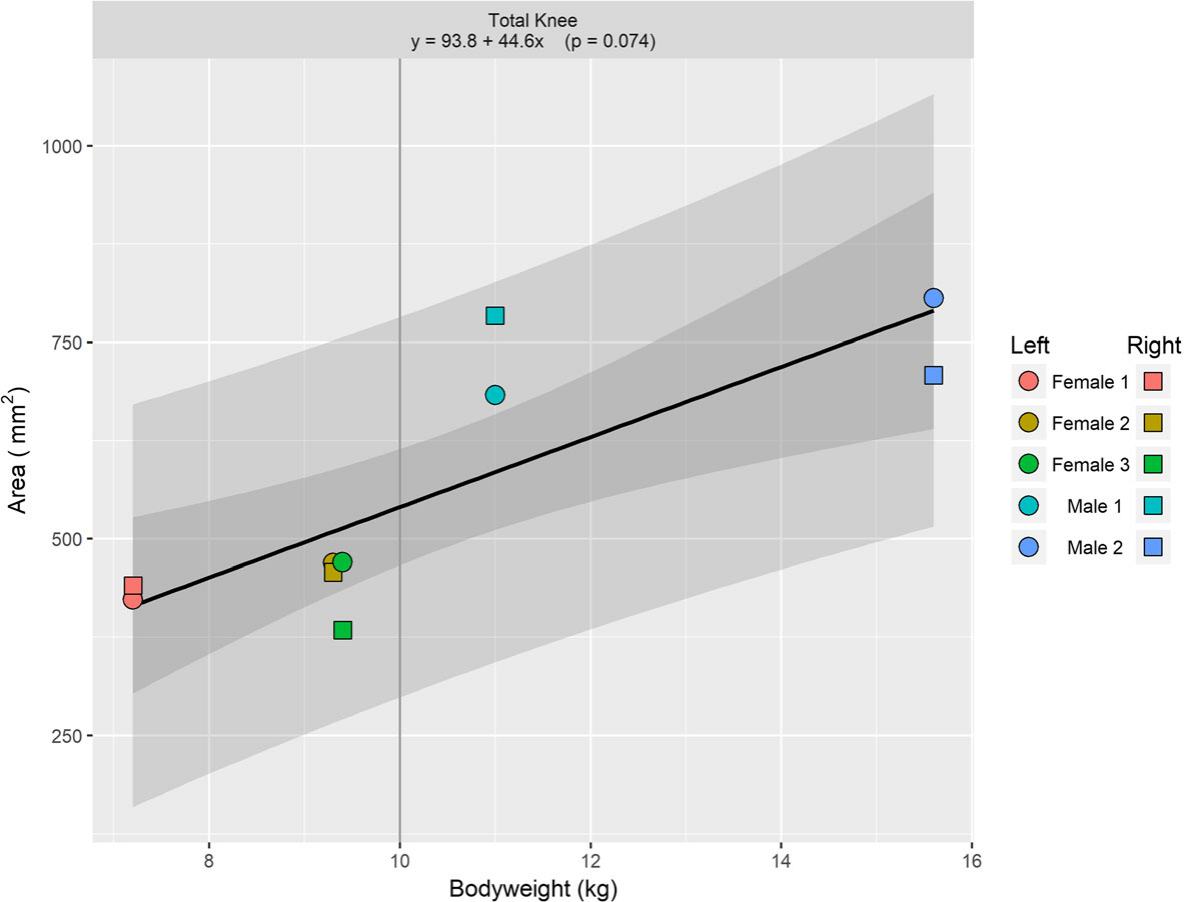
Animal bodyweights regressed against total knee surface area. Data are presented from both knees from each animal, where available. The regression equation from a simple linear model of surface area versus bodyweight is given in each panel header, along with the p-value for the gradient. The lighter shaded region shows the 95% prediction interval for this model, illustrating the range of plausible future values under this model; the darker shaded region shows the 95% confidence interval for the regression line

Mean knee cartilage volume, averaged across the right and left knees, ranged from 319.7mm^3^ to 647.3 mm^3^; while the mean knee cartilage surface area ranged from 427.1 mm^2^ to 757.2 mm^2^. Figures 1 and 2 show the relationships between animal bodyweight and the volume of various knee compartments and total knee volume; Figs. 3 and 4 show the same relationships for surface areas. All relationships showed a positive correlation between bodyweight and volume or surface area, albeit not all statistically significantly at the 5% level (Fig. 5). In particular, the evidence for medial compartment surface area and volume increasing with increasing bodyweight was weak.

Total knee surface area increased an estimated 44.6mm^2^ per increase in kg of bodyweight, with a 10 kg animal having an estimated total knee surface area of approximately 540mm^2^; and total knee volume increased an estimated 43.7mm^2^ per increase in kg of bodyweight, with a 10 kg animal having an estimated total knee volume of approximately 416mm^3^. Both relationships were statistically significant at the 5% level, with *p*-values of 0.004 and <0.001 respectively.

Cartilage thickness could be approximated by assuming everything is a sphere and calculating the radii based on surface area and volume calculations. Doing so shows that cartilage thickness also increases with increasing bodyweight with thickness ranging from 1.75 mm to 2.5 mm.

## Discussion

This study has provided important information as to the cartilage volume and surface area of the Beagle dog that can be utilized as a reference value for future pre-clinical trials utilizing canine models for the investigation of OA and related conditions.

A combination of pre-clinical and clinical studies has resulted in the current knowledge state in regards to OA pathogenesis and therapeutic efficacy of treatment modalities (Bendele 2001; Lampropoulou-Adamidou et al. 2014 **)**. Despite their usefulness, human clinical studies have several limitations. Variation in disease initiation, symptom severity and disease progression make it very challenging to precisely study OA in humans (Matthews 2013; Karsdal et al. 2014). Without preclinical models, the restrictions of clinical trials would have inhibited current medical advances in understanding and treating OA.

Both surgically induced and naturally occurring canine models of OA have been extensively studied as this species is thought by some to be the closest to a gold standard model. This is because of the existing similarities in anatomy, disease progression and translation of outcomes to humans (Gregory et al. 2012; Marijnissen et al. 2002; Moreau et al. 2013). Important differences in biomechanics and gait do exist, particularly in regards to the much greater knee flexion angle in the dog (Proffen et al. 2012). However, the ability to modulate exercise regimens/rehabilitation for the animals, modify their weight bearing status and provide longitudinal outcome measures make this model attractive for DMOAD investigation.

Partially due to the common presentation of clinical OA in dogs, a variety of antemortem diagnostic monitoring protocols have been designed, several of which have been used in several experimental models. These include gait and kinematic analyses, as well as imaging techniques (Matyas et al. 2013; Moreau et al. 2013). Of the latter, MRI in particular has long been of interest because of its ability to accurately image cartilage and other joint structures longitudinally. It is known that dog cartilage is slightly less than half the thickness of human cartilage (Ahern et al. 2009). However, data concerning canine cartilage volume and surface area is scarce.

The data presented in this study is potentially valuable. When monitoring cartilage changes over time, or evaluating the efficacy of therapeutic agents on the disease process, the inter-individual variability can be neglected since comparative values for each individual at different points in time will exist. However, when trying to retrospectively estimate the amount of cartilage loss at the onset of clinical symptoms, the measurements at a given time point need to be related to the normal values in an appropriate reference population, or some estimate of the original state of cartilage in the given species (Eckstein et al. 1998; Eckstein et al. 2001). Furthermore, experimental dose levels of new therapies (DMOADs) can be justified based on these presented cartilage volumes. Therefore, as a first step we have assessed the normal values for cartilage volume and surface area in Beagle dogs without cartilage damage. Boileau et al. measured a cartilage volume of 1080.0 mm^3^ in five crossbred dogs with a mean weight of 25.0 kg (Boileau et al. 2008); from our regressions, we estimate that 25 kg dogs would have knee cartilage volumes of approximately 1071.0 mm^3^.

Surprisingly, human knee joint cartilage volumes are not associated with the body weight. One may expect that taller individuals would have larger joints and hence more cartilage, and also that a greater body weight would cause an increase in cartilage volume to withstand the heavier load carried. However, this has been shown to not be correct (Eckstein et al. 1998; Eckstein et al. 2001). In contrast, as shown in this study, heavier dogs do tend towards larger cartilage volumes and surface area. Also cartilage thickness tends to increase with increasing bodyweight.

In drawing conclusions it is important to note that the three lightest animals were female, and the three heaviest male. It may be that some of the differences observed are gender related, and the bodyweight effects are driven extrinsically through those gender differences. Given the size of this study these effects cannot be drawn out here, but may warrant further investigation. Also one of the female dogs was only 10 months old and was likely not mature at the time of the MRI evaluation. Another drawback is the fact that cartilage thickness was not directly measured but approximated by assuming everything is a sphere and calculating the radii based on surface area and volume calculations.

A final point of note apparent from review of the graphical presentations is that, although in the majority of cases, animal-to-animal variability is greater than that seen within-animal, there are instances of substantial differences between measurements within the same animal. We would consider it prudent in future studies to continue to examine multiple legs within a single animal; or in longitudinal studies to repeat measurements within the same leg.

An obvious limitation of this study is that the sample size was relatively small with one animal’s cartilage surface area and volume of the tibial aspect of the knee not being estimated due to insufficient data to perform adequate 3D reconstructions of this section. Also the FOV of the MRI imaging was quite large in this study and should be changed in future projects.

However, this pilot study was not intended to be a hypothesis testing study, but rather a small pilot hypothesis generating study delivering a preliminary examination. Future investigations with higher sample sizes could provide more detailed data on the normal cartilage volumes and areas of the Beagle knee joint within various age ranges.

## Conclusions

This study investigated cartilage volumes and areas in Beagle dogs for the first time. We found that body knee volumes and surface areas increased with increasing animal bodyweight, with simple linear relationships. These relationships can act as references for future studies utilizing Beagle dogs for the investigation of cartilage related pathology.
